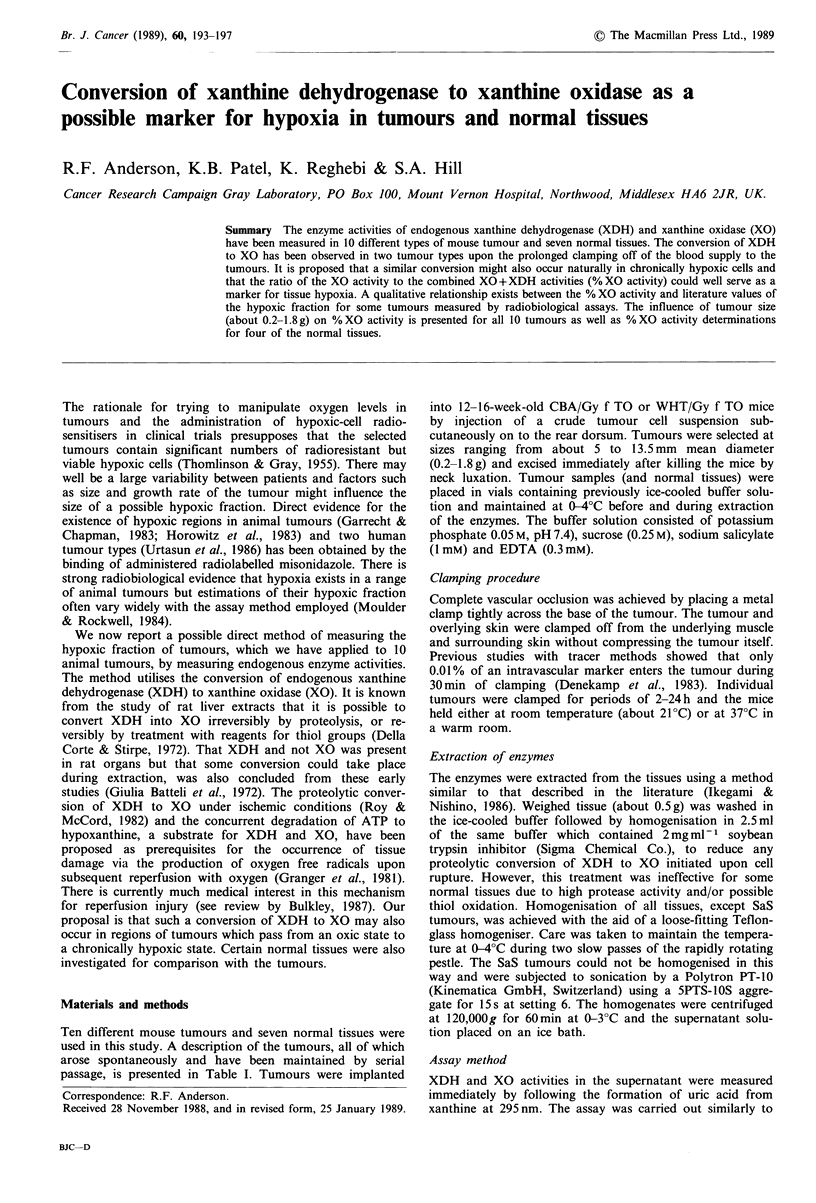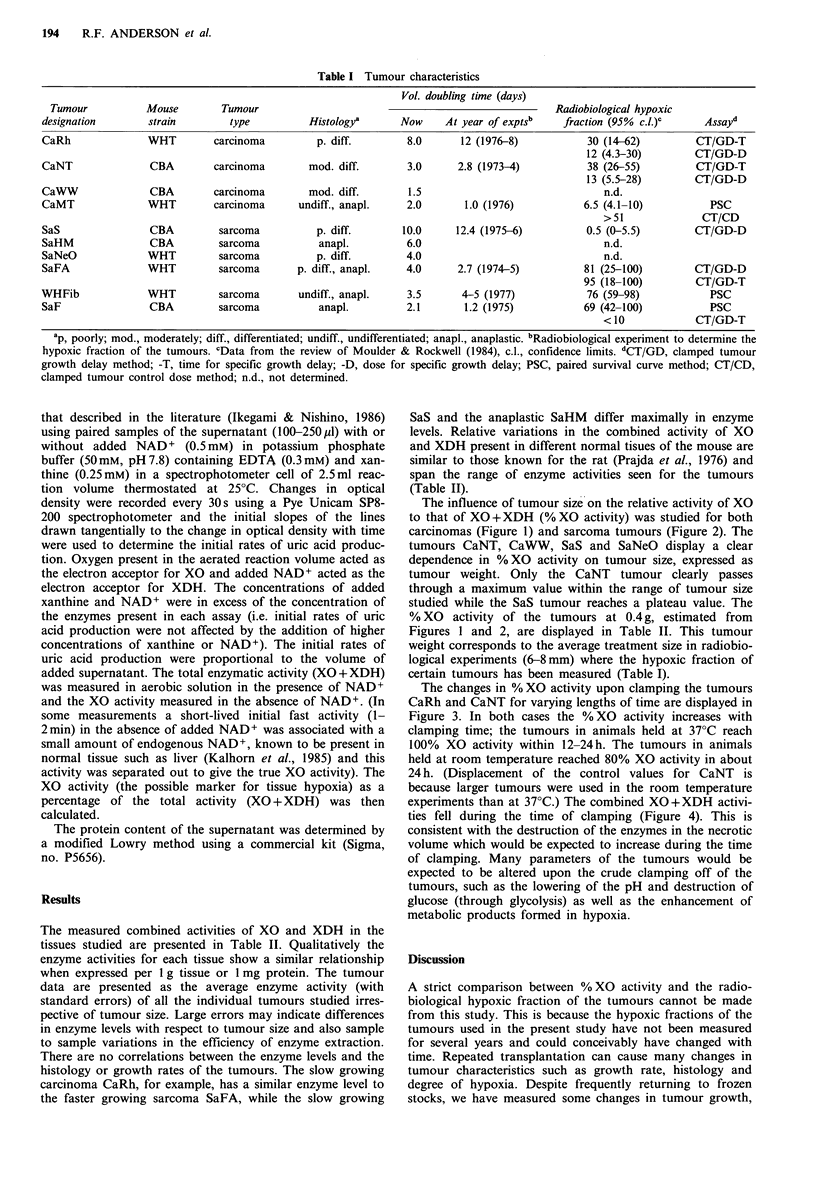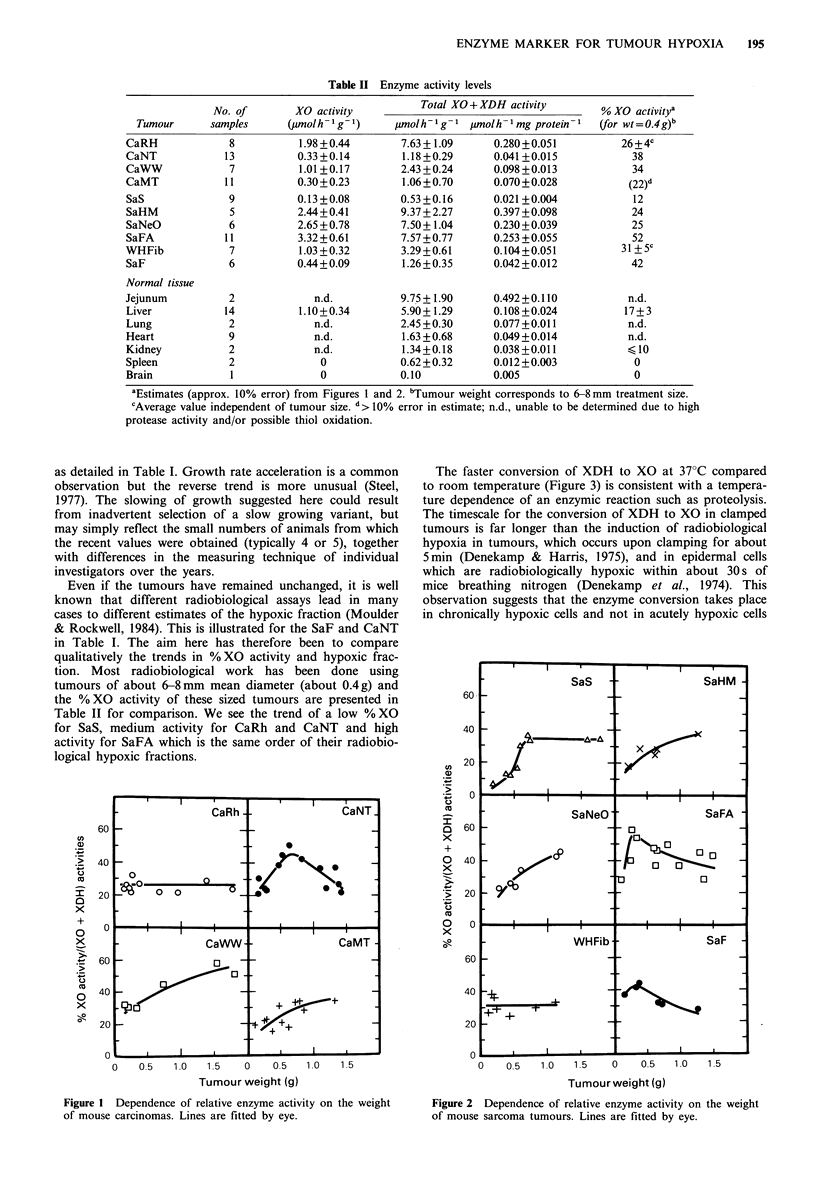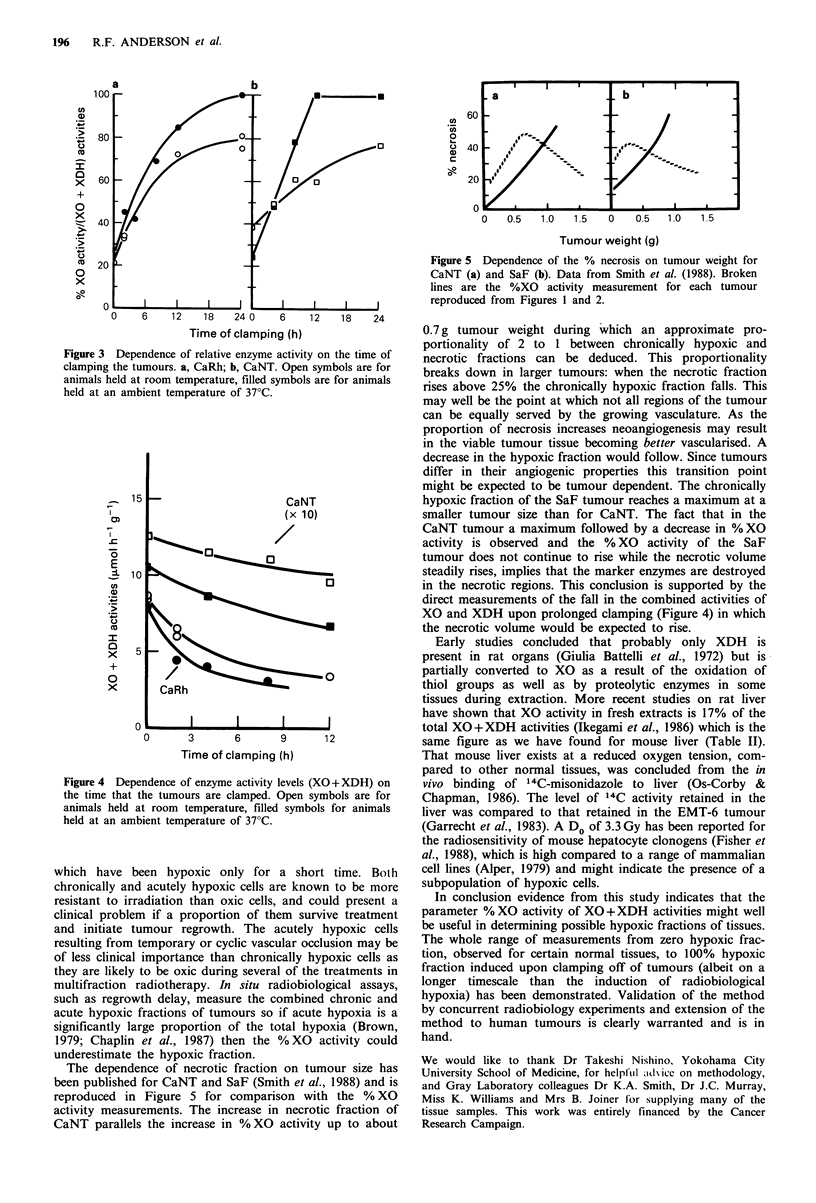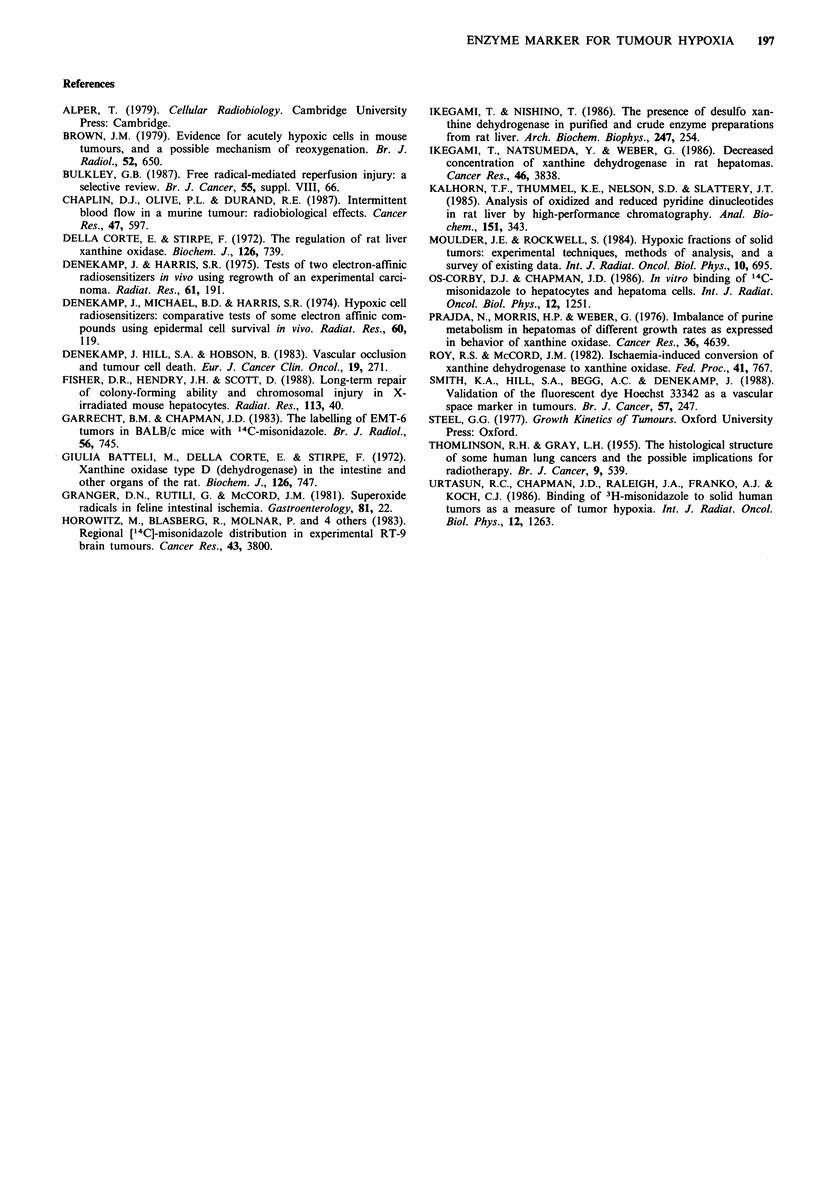# Conversion of xanthine dehydrogenase to xanthine oxidase as a possible marker for hypoxia in tumours and normal tissues.

**DOI:** 10.1038/bjc.1989.249

**Published:** 1989-08

**Authors:** R. F. Anderson, K. B. Patel, K. Reghebi, S. A. Hill

**Affiliations:** Cancer Research Campaign Gray Laboratory, Mount Vernon Hospital, Northwood, Middlesex, UK.

## Abstract

The enzyme activities of endogenous xanthine dehydrogenase (XDH) and xanthine oxidase (XO) have been measured in 10 different types of mouse tumour and seven normal tissues. The conversion of XDH to XO has been observed in two tumour types upon the prolonged clamping off of the blood supply to the tumours. It is proposed that a similar conversion might also occur naturally in chronically hypoxic cells and that the ratio of the XO activity to the combined XO + XDH activities (%XO activity) could well serve as a marker for tissue hypoxia. A qualitative relationship exists between the %XO activity and literature values of the hypoxic fraction for some tumours measured by radiobiological assays. The influence of tumour size (about 0.2-1.8 g) on %XO activity is presented for all 10 tumours as well as %XO activity determinations for four of the normal tissues.


Br. J. Cancer (1989), 60, 193–197 © The Macmillan Press Ltd., 1989

### Conversion of xanthine dehydrogenase to xanthine oxidase as a possible marker for hypoxia in tumours and normal tissues

R.F. Anderson, K.B. Patel, K. Reghebi & S.A. Hill

*Cancer Research Campaign Gray Laboratory, PO Box 100, Mount Vernon Hospital, Northwood, Middlesex HA6 2JR, UK.*

**Summary** The enzyme activities of endogenous xanthine dehydrogenase (XDH) and xanthine oxidase (XO) have been measured in 10 different types of mouse tumour and seven normal tissues. The conversion of XDH to XO has been observed in two tumour types upon the prolonged clamping off of the blood supply to the tumours. It is proposed that a similar conversion might also occur naturally in chronically hypoxic cells and that the ratio of the XO activity to the combined XO+XDH activities (% XO activity) could well serve as a marker for tissue hypoxia. A qualitative relationship exists between the % XO activity and literature values of the hypoxic fraction for some tumours measured by radiobiological assays. The influence of tumour size (about 0.2–1.8 g) on % XO activity is presented for all 10 tumours as well as % XO activity determinations for four of the normal tissues.

The rationale for trying to manipulate oxygen levels in tumours and the administration of hypoxic-cell radiosensitisers in clinical trials presupposes that the selected tumours contain significant numbers of radioresistant but viable hypoxic cells (Thomlinson & Gray, 1955). There may well be a large variability between patients and factors such as size and growth rate of the tumour might influence the size of a possible hypoxic fraction. Direct evidence for the existence of hypoxic regions in animal tumours (Garrecht & Chapman, 1983; Horowitz *et al.*, 1983) and two human tumour types (Urtasun *et al.*, 1986) has been obtained by the binding of administered radiolabelled misonidazole. There is strong radiobiological evidence that hypoxia exists in a range of animal tumours but estimations of their hypoxic fraction often vary widely with the assay method employed (Moulder & Rockwell, 1984).

We now report a possible direct method of measuring the hypoxic fraction of tumours, which we have applied to 10 animal tumours, by measuring endogenous enzyme activities. The method utilises the conversion of endogenous xanthine dehydrogenase (XDH) to xanthine oxidase (XO). It is known from the study of rat liver extracts that it is possible to convert XDH into XO irreversibly by proteolysis, or reversibly by treatment with reagents for thiol groups (Della Corte & Stirpe, 1972). That XDH and not XO was present in rat organs but that some conversion could take place during extraction, was also concluded from these early studies (Giulia Batteli *et al.*, 1972). The proteolytic conversion of XDH to XO under ischemic conditions (Roy & McCord, 1982) and the concurrent degradation of ATP to hypoxanthine, a substrate for XDH and XO, have been proposed as prerequisites for the occurrence of tissue damage via the production of oxygen free radicals upon subsequent reperfusion with oxygen (Granger *et al.*, 1981). There is currently much medical interest in this mechanism for reperfusion injury (see review by Bulkley, 1987). Our proposal is that such a conversion of XDH to XO may also occur in regions of tumours which pass from an oxic state to a chronically hypoxic state. Certain normal tissues were also investigated for comparison with the tumours.

### Materials and methods

Ten different mouse tumours and seven normal tissues were used in this study. A description of the tumours, all of which arose spontaneously and have been maintained by serial passage, is presented in Table I. Tumours were implanted into 12–16-week-old CBA/Gy f TO or WHT/Gy f TO mice by injection of a crude tumour cell suspension subcutaneously on to the rear dorsum. Tumours were selected at sizes ranging from about 5 to 13.5 mm mean diameter (0.2–1.8 g) and excised immediately after killing the mice by neck luxation. Tumour samples (and normal tissues) were placed in vials containing previously ice-cooled buffer solution and maintained at 0–4°C before and during extraction of the enzymes. The buffer solution consisted of potassium phosphate 0.05 M, pH 7.4), sucrose (0.25 M), sodium salicylate (1 mM) and EDTA (0.3 mM).

*Clamping procedure*

Complete vascular occlusion was achieved by placing a metal clamp tightly across the base of the tumour. The tumour and overlying skin were clamped off from the underlying muscle and surrounding skin without compressing the tumour itself. Previous studies with tracer methods showed that only 0.01% of an intravascular marker enters the tumour during 30 min of clamping (Denekamp *et al.*, 1983). Individual tumours were clamped for periods of 2–24 h and the mice held either at room temperature (about 21°C) or at 37°C in a warm room.

*Extraction of enzymes*

The enzymes were extracted from the tissues using a method similar to that described in the literature (Ikegami & Nishino, 1986). Weighed tissue (about 0.5 g) was washed in the ice-cooled buffer followed by homogenisation in 2.5 ml of the same buffer which contained 2 mg ml$^{-1}$ soybean trypsin inhibitor (Sigma Chemical Co.), to reduce any proteolytic conversion of XDH to XO initiated upon cell rupture. However, this treatment was ineffective for some normal tissues due to high protease activity and/or possible thiol oxidation. Homogenisation of all tissues, except SaS tumours, was achieved with the aid of a loose-fitting Teflon-glass homogeniser. Care was taken to maintain the temperature at 0–4°C during two slow passes of the rapidly rotating pestle. The SaS tumours could not be homogenised in this way and were subjected to sonication by a Polytron PT-10 (Kinematica GmbH, Switzerland) using a 5PTS-10S aggregate for 15 s at setting 6. The homogenates were centrifuged at 120,000 *g* for 60 min at 0–3°C and the supernatant solution placed on an ice bath.

*Assay method*

XDH and XO activities in the supernatant were measured immediately by following the formation of uric acid from xanthine at 295 nm. The assay was carried out similarly to

Correspondence: R.F. Anderson.
Received 28 November 1988, and in revised form, 25 January 1989.

**Table I** Tumour characteristics

| *Tumour designation* | *Mouse strain* | *Tumour type* | *Histology*[a] | *Vol. doubling time (days)* Now | *Vol. doubling time (days)* At year of expts[b] | *Radiobiological hypoxic fraction (95% c.l.)*[c] | *Assay*[d] |
|---|---|---|---|---|---|---|---|
| CaRh | WHT | carcinoma | p. diff. | 8.0 | 12 (1976–8) | 30 (14–62) | CT/GD-T |
| | | | | | | 12 (4.3–30) | CT/GD-D |
| CaNT | CBA | carcinoma | mod. diff. | 3.0 | 2.8 (1973–4) | 38 (26–55) | CT/GD-T |
| | | | | | | 13 (5.5–28) | CT/GD-D |
| CaWW | CBA | carcinoma | mod. diff. | 1.5 | | n.d. | |
| CaMT | WHT | carcinoma | undiff., anapl. | 2.0 | 1.0 (1976) | 6.5 (4.1–10) | PSC |
| | | | | | | >51 | CT/CD |
| SaS | CBA | sarcoma | p. diff. | 10.0 | 12.4 (1975–6) | 0.5 (0–5.5) | CT/GD-D |
| SaHM | CBA | sarcoma | anapl. | 6.0 | | n.d. | |
| SaNeO | WHT | sarcoma | p. diff. | 4.0 | | n.d. | |
| SaFA | WHT | sarcoma | p. diff., anapl. | 4.0 | 2.7 (1974–5) | 81 (25–100) | CT/GD-D |
| | | | | | | 95 (18–100) | CT/GD-T |
| WHFib | WHT | sarcoma | undiff., anapl. | 3.5 | 4–5 (1977) | 76 (59–98) | PSC |
| SaF | CBA | sarcoma | anapl. | 2.1 | 1.2 (1975) | 69 (42–100) | PSC |
| | | | | | | <10 | CT/GD-T |

[a]p, poorly; mod., moderately; diff., differentiated; undiff., undifferentiated; anapl., anaplastic. [b]Radiobiological experiment to determine the hypoxic fraction of the tumours. [c]Data from the review of Moulder & Rockwell (1984), c.l., confidence limits. [d]CT/GD, clamped tumour growth delay method; -T, time for specific growth delay; -D, dose for specific growth delay; PSC, paired survival curve method; CT/CD, clamped tumour control dose method; n.d., not determined.

that described in the literature (Ikegami & Nishino, 1986) using paired samples of the supernatant (100–250 $\mu$l) with or without added $NAD^+$ (0.5 mM) in potassium phosphate buffer (50 mM, pH 7.8) containing EDTA (0.3 mM) and xanthine (0.25 mM) in a spectrophotometer cell of 2.5 ml reaction volume thermostated at 25°C. Changes in optical density were recorded every 30 s using a Pye Unicam SP8-200 spectrophotometer and the initial slopes of the lines drawn tangentially to the change in optical density with time were used to determine the initial rates of uric acid production. Oxygen present in the aerated reaction volume acted as the electron acceptor for XO and added $NAD^+$ acted as the electron acceptor for XDH. The concentrations of added xanthine and $NAD^+$ were in excess of the concentration of the enzymes present in each assay (i.e. initial rates of uric acid production were not affected by the addition of higher concentrations of xanthine or $NAD^+$). The initial rates of uric acid production were proportional to the volume of added supernatant. The total enzymatic activity (XO + XDH) was measured in aerobic solution in the presence of $NAD^+$ and the XO activity measured in the absence of $NAD^+$. (In some measurements a short-lived initial fast activity (1–2 min) in the absence of added $NAD^+$ was associated with a small amount of endogenous $NAD^+$, known to be present in normal tissue such as liver (Kalhorn *et al.*, 1985) and this activity was separated out to give the true XO activity). The XO activity (the possible marker for tissue hypoxia) as a percentage of the total activity (XO + XDH) was then calculated.

The protein content of the supernatant was determined by a modified Lowry method using a commercial kit (Sigma, no. P5656).

### Results

The measured combined activities of XO and XDH in the tissues studied are presented in Table II. Qualitatively the enzyme activities for each tissue show a similar relationship when expressed per 1 g tissue or 1 mg protein. The tumour data are presented as the average enzyme activity (with standard errors) of all the individual tumours studied irrespective of tumour size. Large errors may indicate differences in enzyme levels with respect to tumour size and also sample to sample variations in the efficiency of enzyme extraction. There are no correlations between the enzyme levels and the histology or growth rates of the tumours. The slow growing carcinoma CaRh, for example, has a similar enzyme level to the faster growing sarcoma SaFA, while the slow growing SaS and the anaplastic SaHM differ maximally in enzyme levels. Relative variations in the combined activity of XO and XDH present in different normal tisues of the mouse are similar to those known for the rat (Prajda *et al.*, 1976) and span the range of enzyme activities seen for the tumours (Table II).

The influence of tumour size on the relative activity of XO to that of XO + XDH (% XO activity) was studied for both carcinomas (Figure 1) and sarcoma tumours (Figure 2). The tumours CaNT, CaWW, SaS and SaNeO display a clear dependence in % XO activity on tumour size, expressed as tumour weight. Only the CaNT tumour clearly passes through a maximum value within the range of tumour size studied while the SaS tumour reaches a plateau value. The % XO activity of the tumours at 0.4 g, estimated from Figures 1 and 2, are displayed in Table II. This tumour weight corresponds to the average treatment size in radiobiological experiments (6–8 mm) where the hypoxic fraction of certain tumours has been measured (Table I).

The changes in % XO activity upon clamping the tumours CaRh and CaNT for varying lengths of time are displayed in Figure 3. In both cases the % XO activity increases with clamping time; the tumours in animals held at 37°C reach 100% XO activity within 12–24 h. The tumours in animals held at room temperature reached 80% XO activity in about 24 h. (Displacement of the control values for CaNT is because larger tumours were used in the room temperature experiments than at 37°C.) The combined XO + XDH activities fell during the time of clamping (Figure 4). This is consistent with the destruction of the enzymes in the necrotic volume which would be expected to increase during the time of clamping. Many parameters of the tumours would be expected to be altered upon the crude clamping off of the tumours, such as the lowering of the pH and destruction of glucose (through glycolysis) as well as the enhancement of metabolic products formed in hypoxia.

### Discussion

A strict comparison between % XO activity and the radiobiological hypoxic fraction of the tumours cannot be made from this study. This is because the hypoxic fractions of the tumours used in the present study have not been measured for several years and could conceivably have changed with time. Repeated transplantation can cause many changes in tumour characteristics such as growth rate, histology and degree of hypoxia. Despite frequently returning to frozen stocks, we have measured some changes in tumour growth,

**Table II** Enzyme activity levels

| Tumour | No. of samples | XO activity ($\mu mol\,h^{-1}\,g^{-1}$) | Total XO+XDH activity | | % XO activity[a] (for wt=0.4 g)[b] |
|---|---|---|---|---|---|
| | | | $\mu mol\,h^{-1}\,g^{-1}$ | $\mu mol\,h^{-1}$ mg protein$^{-1}$ | |
| CaRH | 8 | 1.98±0.44 | 7.63±1.09 | 0.280±0.051 | 26±4[c] |
| CaNT | 13 | 0.33±0.14 | 1.18±0.29 | 0.041±0.015 | 38 |
| CaWW | 7 | 1.01±0.17 | 2.43±0.24 | 0.098±0.013 | 34 |
| CaMT | 11 | 0.30±0.23 | 1.06±0.70 | 0.070±0.028 | (22)[d] |
| SaS | 9 | 0.13±0.08 | 0.53±0.16 | 0.021±0.004 | 12 |
| SaHM | 5 | 2.44±0.41 | 9.37±2.27 | 0.397±0.098 | 24 |
| SaNeO | 6 | 2.65±0.78 | 7.50±1.04 | 0.230±0.039 | 25 |
| SaFA | 11 | 3.32±0.61 | 7.57±0.77 | 0.253±0.055 | 52 |
| WHFib | 7 | 1.03±0.32 | 3.29±0.61 | 0.104±0.051 | 31±5[c] |
| SaF | 6 | 0.44±0.09 | 1.26±0.35 | 0.042±0.012 | 42 |
| *Normal tissue* | | | | | |
| Jejunum | 2 | n.d. | 9.75±1.90 | 0.492±0.110 | n.d. |
| Liver | 14 | 1.10±0.34 | 5.90±1.29 | 0.108±0.024 | 17±3 |
| Lung | 2 | n.d. | 2.45±0.30 | 0.077±0.011 | n.d. |
| Heart | 9 | n.d. | 1.63±0.68 | 0.049±0.014 | n.d. |
| Kidney | 2 | n.d. | 1.34±0.18 | 0.038±0.011 | ≤10 |
| Spleen | 2 | 0 | 0.62±0.32 | 0.012±0.003 | 0 |
| Brain | 1 | 0 | 0.10 | 0.005 | 0 |

[a]Estimates (approx. 10% error) from Figures 1 and 2. [b]Tumour weight corresponds to 6–8 mm treatment size. [c]Average value independent of tumour size. [d]> 10% error in estimate; n.d., unable to be determined due to high protease activity and/or possible thiol oxidation.

as detailed in Table I. Growth rate acceleration is a common observation but the reverse trend is more unusual (Steel, 1977). The slowing of growth suggested here could result from inadvertent selection of a slow growing variant, but may simply reflect the small numbers of animals from which the recent values were obtained (typically 4 or 5), together with differences in the measuring technique of individual investigators over the years.

Even if the tumours have remained unchanged, it is well known that different radiobiological assays lead in many cases to different estimates of the hypoxic fraction (Moulder & Rockwell, 1984). This is illustrated for the SaF and CaNT in Table I. The aim here has therefore been to compare qualitatively the trends in % XO activity and hypoxic fraction. Most radiobiological work has been done using tumours of about 6–8 mm mean diameter (about 0.4 g) and the % XO activity of these sized tumours are presented in Table II for comparison. We see the trend of a low % XO for SaS, medium activity for CaRh and CaNT and high activity for SaFA which is the same order of their radiobiological hypoxic fractions.

**Figure 1** Dependence of relative enzyme activity on the weight of mouse carcinomas. Lines are fitted by eye.

The faster conversion of XDH to XO at 37°C compared to room temperature (Figure 3) is consistent with a temperature dependence of an enzymic reaction such as proteolysis. The timescale for the conversion of XDH to XO in clamped tumours is far longer than the induction of radiobiological hypoxia in tumours, which occurs upon clamping for about 5 min (Denekamp & Harris, 1975), and in epidermal cells which are radiobiologically hypoxic within about 30 s of mice breathing nitrogen (Denekamp *et al.*, 1974). This observation suggests that the enzyme conversion takes place in chronically hypoxic cells and not in acutely hypoxic cells

**Figure 2** Dependence of relative enzyme activity on the weight of mouse sarcoma tumours. Lines are fitted by eye.

**Figure 3** Dependence of relative enzyme activity on the time of clamping the tumours. **a**, CaRh; **b**, CaNT. Open symbols are for animals held at room temperature, filled symbols are for animals held at an ambient temperature of 37°C.

**Figure 4** Dependence of enzyme activity levels (XO+XDH) on the time that the tumours are clamped. Open symbols are for animals held at room temperature, filled symbols for animals held at an ambient temperature of 37°C.

which have been hypoxic only for a short time. Both chronically and acutely hypoxic cells are known to be more resistant to irradiation than oxic cells, and could present a clinical problem if a proportion of them survive treatment and initiate tumour regrowth. The acutely hypoxic cells resulting from temporary or cyclic vascular occlusion may be of less clinical importance than chronically hypoxic cells as they are likely to be oxic during several of the treatments in multifraction radiotherapy. *In situ* radiobiological assays, such as regrowth delay, measure the combined chronic and acute hypoxic fractions of tumours so if acute hypoxia is a significantly large proportion of the total hypoxia (Brown, 1979; Chaplin *et al.*, 1987) then the %XO activity could underestimate the hypoxic fraction.

The dependence of necrotic fraction on tumour size has been published for CaNT and SaF (Smith *et al.*, 1988) and is reproduced in Figure 5 for comparison with the %XO activity measurements. The increase in necrotic fraction of CaNT parallels the increase in %XO activity up to about

**Figure 5** Dependence of the % necrosis on tumour weight for CaNT (**a**) and SaF (**b**). Data from Smith *et al.* (1988). Broken lines are the %XO activity measurement for each tumour reproduced from Figures 1 and 2.

0.7 g tumour weight during which an approximate proportionality of 2 to 1 between chronically hypoxic and necrotic fractions can be deduced. This proportionality breaks down in larger tumours: when the necrotic fraction rises above 25% the chronically hypoxic fraction falls. This may well be the point at which not all regions of the tumour can be equally served by the growing vasculature. As the proportion of necrosis increases neoangiogenesis may result in the viable tumour tissue becoming *better* vascularised. A decrease in the hypoxic fraction would follow. Since tumours differ in their angiogenic properties this transition point might be expected to be tumour dependent. The chronically hypoxic fraction of the SaF tumour reaches a maximum at a smaller tumour size than for CaNT. The fact that in the CaNT tumour a maximum followed by a decrease in %XO activity is observed and the %XO activity of the SaF tumour does not continue to rise while the necrotic volume steadily rises, implies that the marker enzymes are destroyed in the necrotic regions. This conclusion is supported by the direct measurements of the fall in the combined activities of XO and XDH upon prolonged clamping (Figure 4) in which the necrotic volume would be expected to rise.

Early studies concluded that probably only XDH is present in rat organs (Giulia Battelli *et al.*, 1972) but is partially converted to XO as a result of the oxidation of thiol groups as well as by proteolytic enzymes in some tissues during extraction. More recent studies on rat liver have shown that XO activity in fresh extracts is 17% of the total XO+XDH activities (Ikegami *et al.*, 1986) which is the same figure as we have found for mouse liver (Table II). That mouse liver exists at a reduced oxygen tension, compared to other normal tissues, was concluded from the *in vivo* binding of $^{14}$C-misonidazole to liver (Os-Corby & Chapman, 1986). The level of $^{14}$C activity retained in the liver was compared to that retained in the EMT-6 tumour (Garrecht *et al.*, 1983). A $D_0$ of 3.3 Gy has been reported for the radiosensitivity of mouse hepatocyte clonogens (Fisher *et al.*, 1988), which is high compared to a range of mammalian cell lines (Alper, 1979) and might indicate the presence of a subpopulation of hypoxic cells.

In conclusion evidence from this study indicates that the parameter %XO activity of XO+XDH activities might well be useful in determining possible hypoxic fractions of tissues. The whole range of measurements from zero hypoxic fraction, observed for certain normal tissues, to 100% hypoxic fraction induced upon clamping off of tumours (albeit on a longer timescale than the induction of radiobiological hypoxia) has been demonstrated. Validation of the method by concurrent radiobiology experiments and extension of the method to human tumours is clearly warranted and is in hand.

We would like to thank Dr Takeshi Nishino, Yokohama City University School of Medicine, for helpful advice on methodology, and Gray Laboratory colleagues Dr K.A. Smith, Dr J.C. Murray, Miss K. Williams and Mrs B. Joiner for supplying many of the tissue samples. This work was entirely financed by the Cancer Research Campaign.

**References**

ALPER, T. (1979). *Cellular Radiobiology*. Cambridge University Press: Cambridge.

BROWN, J.M. (1979). Evidence for acutely hypoxic cells in mouse tumours, and a possible mechanism of reoxygenation. *Br. J. Radiol.*, **52**, 650.

BULKLEY, G.B. (1987). Free radical-mediated reperfusion injury: a selective review. *Br. J. Cancer*, **55**, suppl. VIII, 66.

CHAPLIN, D.J., OLIVE, P.L. & DURAND, R.E. (1987). Intermittent blood flow in a murine tumour: radiobiological effects. *Cancer Res.*, **47**, 597.

DELLA CORTE, E. & STIRPE, F. (1972). The regulation of rat liver xanthine oxidase. *Biochem. J.*, **126**, 739.

DENEKAMP, J. & HARRIS, S.R. (1975). Tests of two electron-affinic radiosensitizers *in vivo* using regrowth of an experimental carcinoma. *Radiat. Res.*, **61**, 191.

DENEKAMP, J., MICHAEL, B.D. & HARRIS, S.R. (1974). Hypoxic cell radiosensitizers: comparative tests of some electron affinic compounds using epidermal cell survival *in vivo*. *Radiat. Res.*, **60**, 119.

DENEKAMP, J. HILL, S.A. & HOBSON, B. (1983). Vascular occlusion and tumour cell death. *Eur. J. Cancer Clin. Oncol.*, **19**, 271.

FISHER, D.R., HENDRY, J.H. & SCOTT, D. (1988). Long-term repair of colony-forming ability and chromosomal injury in X-irradiated mouse hepatocytes. *Radiat. Res.*, **113**, 40.

GARRECHT, B.M. & CHAPMAN, J.D. (1983). The labelling of EMT-6 tumors in BALB/c mice with $^{14}$C-misonidazole. *Br. J. Radiol.*, **56**, 745.

GIULIA BATTELI, M., DELLA CORTE, E. & STIRPE, F. (1972). Xanthine oxidase type D (dehydrogenase) in the intestine and other organs of the rat. *Biochem. J.*, **126**, 747.

GRANGER, D.N., RUTILI, G. & McCORD, J.M. (1981). Superoxide radicals in feline intestinal ischemia. *Gastroenterology*, **81**, 22.

HOROWITZ, M., BLASBERG, R., MOLNAR, P. and 4 others (1983). Regional [$^{14}$C]-misonidazole distribution in experimental RT-9 brain tumours. *Cancer Res.*, **43**, 3800.

IKEGAMI, T. & NISHINO, T. (1986). The presence of desulfo xanthine dehydrogenase in purified and crude enzyme preparations from rat liver. *Arch. Biochem. Biophys.*, **247**, 254.

IKEGAMI, T., NATSUMEDA, Y. & WEBER, G. (1986). Decreased concentration of xanthine dehydrogenase in rat hepatomas. *Cancer Res.*, **46**, 3838.

KALHORN, T.F., THUMMEL, K.E., NELSON, S.D. & SLATTERY, J.T. (1985). Analysis of oxidized and reduced pyridine dinucleotides in rat liver by high-performance chromatography. *Anal. Biochem.*, **151**, 343.

MOULDER, J.E. & ROCKWELL, S. (1984). Hypoxic fractions of solid tumors: experimental techniques, methods of analysis, and a survey of existing data. *Int. J. Radiat. Oncol. Biol. Phys.*, **10**, 695.

OS-CORBY, D.J. & CHAPMAN, J.D. (1986). *In vitro* binding of $^{14}$C-misonidazole to hepatocytes and hepatoma cells. *Int. J. Radiat. Oncol. Biol. Phys.*, **12**, 1251.

PRAJDA, N., MORRIS, H.P. & WEBER, G. (1976). Imbalance of purine metabolism in hepatomas of different growth rates as expressed in behavior of xanthine oxidase. *Cancer Res.*, **36**, 4639.

ROY, R.S. & McCORD, J.M. (1982). Ischaemia-induced conversion of xanthine dehydrogenase to xanthine oxidase. *Fed. Proc.*, **41**, 767.

SMITH, K.A., HILL, S.A., BEGG, A.C. & DENEKAMP, J. (1988). Validation of the fluorescent dye Hoechst 33342 as a vascular space marker in tumours. *Br. J. Cancer*, **57**, 247.

STEEL, G.G. (1977). *Growth Kinetics of Tumours*. Oxford University Press: Oxford.

THOMLINSON, R.H. & GRAY, L.H. (1955). The histological structure of some human lung cancers and the possible implications for radiotherapy. *Br. J. Cancer*, **9**, 539.

URTASUN, R.C., CHAPMAN, J.D., RALEIGH, J.A., FRANKO, A.J. & KOCH, C.J. (1986). Binding of $^{3}$H-misonidazole to solid human tumors as a measure of tumor hypoxia. *Int. J. Radiat. Oncol. Biol. Phys.*, **12**, 1263.